# The role of health economics within health technology assessment: past, present, and future – an Austrian perspective

**DOI:** 10.1017/S0266462324000503

**Published:** 2024-11-05

**Authors:** Ingrid Zechmeister-Koss, Gregor Götz, Daniel Fabian, Claudia Wild

**Affiliations:** Austrian Institute for Health Technology Assessment (AIHTA), Vienna, Austria

**Keywords:** economic evaluation, health economics, methods, societal values, political-economics

## Abstract

In many countries, the economics domain forms a routine part of health technology assessments (HTA) next to analyzing the comparative effectiveness and safety of a technology. The method applied most often is economic evaluation, such as cost-effectiveness analysis, which is supposed to support the efficient use of resources. In Austria, economic evaluation has played a negligible role in HTA and reimbursement decisions, even though the country faces the same public healthcare sustainability challenges as others. In this commentary, we argue that while health economics will need to play a more active role in HTA-related decision support to deal with those challenges, current approaches in other countries may have to be broadened to fit the Austrian context. We are outlining four arguments to underpin this perspective: First, economic evaluations (in their current form) are of limited benefit for supporting reimbursement decisions of new high-priced technologies. Second, a broader variety of health economic methods is needed to address the scope of technologies. Third, applying health economic methods requires a reflection on their underlying values. Finally, health economics within HTA needs to go beyond microeconomic analysis of interventions. We are suggesting several alternative methods and approaches, encouraging out-of-the-box thinking and experimenting with methods developed in the academic context but rarely applied in routine HTA. Although some of our topics are unique to Austria, others may equally apply to other healthcare systems. With our thoughts, we aim to stimulate discussions for further developing health economics within HTA in Austria and internationally.

## Background

In many countries, the economics domain forms a routine part of health technology assessments (HTA) next to analyzing the comparative effectiveness and safety of a technology, with economic evaluations such as cost-effectiveness analysis being the most often used method (1–3). An economic evaluation aims to support an efficient use of resources by comparing new interventions to alternative treatments regarding their costs and outcomes. It results in an incremental cost-effectiveness ratio (ICER), the ratio between the incremental costs and the incremental effects or utilities of the alternatives compared. An ICER below a defined cost-effectiveness threshold indicates that the therapy is cost-effective. In this case, paying for the new treatment suggests that overall, we will gain more population health than we would lose elsewhere by the opportunity forgone to invest in an alternative intervention (opportunity costs). On the contrary, funding a therapy with an ICER above the thresholds indicates that more health will be lost elsewhere on a population level than gained with the new treatment (4).

In contrast to other countries, the domain of health economics has played a minor role in the different units involved in producing HTA in Austria. Within the Austrian Institute for HTA (AIHTA), which is responsible for evaluating medical devices for the hospital benefit catalogue and other technologies, except outpatient drugs, economic studies (economic evaluation, budget impact analysis) represent only 3 percent of all decision-maker-commissioned reports since its existence in 2006 (5). In the assessment of outpatient pharmaceuticals for which the health insurance is responsible, there is a legal requirement for the manufacturer to submit an economic evaluation (called a pharmaco-economic study) as part of the reimbursement application in a limited number of cases, where the marketing authorization holder claims a substantial added therapeutic benefit of the drug (6). Yet, compared to other countries, the significance of the study results in the decision process has been unclear and formal, publicly accessible documents do not specify the role of ICERs as a decision criterion (7). A recently passed law requires selected high-cost or specialized drugs used in hospitals or at the interface between hospitals and outpatient care to undergo an HTA. The law states that the industry can be requested to provide an economic study, mentioning cost-utility analysis (CUA) as an example. However, it does not outline further details on the methods to be used and what role the study results will play in the reimbursement decisions of the products. Contrary to other countries, there is also no mandatory guideline for economic evaluations, defining the methodological standard, available in Austria. Existing documents are outdated and vague. They represent recommendations rather than clear guidance (8).

Still, Austria faces the same healthcare system challenges as other countries, such as struggling with the affordability of new ultrahigh-price interventions, inappropriate use of interventions, waste of resources and maintaining the sustainability of the publicly funded system with access to beneficial technologies for all (9–11). These are all inherently economic topics. As elsewhere, public healthcare resources are limited in Austria, so some form of priority setting and criteria for defining the statutory healthcare benefit package are inevitable (12). Health economics will, therefore, need to play a certain role in Austria’s HTA activities and beyond in the future. By outlining possible future directions for Austria, we intend to stimulate discussions on the future role of health economics and the methods applied within HTA more generally.

## Future prospects for health economics within HTA in Austria

It seems evident that to strengthen the role of health economics within HTA in Austria, efforts are taken toward following international standards, particularly those from EU countries, using study designs recommended in international health economic guidelines. In the remainder of this commentary, we argue that current approaches in other countries may not be the best possible way forward for Austria, and we are reflecting on alternative routes for using economic evidence. We justify our thoughts with four arguments, which we are outlining in the following section.

### Economic evaluations (in their current form) are of limited benefit for supporting reimbursement decisions of new high-priced technologies

As in other countries of the Global North, one of the biggest challenges in the Austrian healthcare systems currently are the novel ultrahigh-price technologies, such as gene therapies. Treatment costs per patient can reach over a million Euros (13), and decision-makers struggle with affordability within limited budgets. As the examples of enzyme replacement therapies (14), gene therapies (15) or new treatments for certain orphan diseases such as spinal muscular atrophy (16) show, ICERs in cost-effectiveness analyses of such therapies are usually very high. In several cases are they beyond one million Euros per QALY (14;16). Obviously, such an ICER is far beyond any national cost-effectiveness threshold, which means that the intervention is not cost-effective. Still, these drugs are publicly funded, at least in high-income countries (13). Systematic funding of interventions despite very unfavorable ICERs indicates that criteria other than efficiency seem to be prioritized. These could, for example, be the severity of a disease or that no treatment alternative is available (13). For Austria with a small HTA budget, we are questioning how useful traditional economic evaluations are in cases where it may be clear that the ICER will be in a very high range (mainly driven by the high prices) but the product will be funded anyway for reasons other than efficiency.

Nevertheless, we think it is important to make Austrian decision-makers aware of the opportunity costs of products with high additional costs in relation to the additional benefits. This includes information that funding such products may mean losing more health elsewhere than what can be gained with the new therapy. Usually, these implications and their consequences for patients remain undebated in Austria because ICERs and opportunity costs are an abstract concept to most decision-makers and other stakeholders involved in pricing and reimbursement processes. Patients whose health is negatively affected by displaced healthcare are invisible, or, as Vallejyo-Torres (17, p. 382) puts it, these are “the patients not in the room.” Methods developed in other countries, such as the opportunity cost calculator (https://bit.ly/4bSgIdy), which shows how many QALYs are lost elsewhere as a result of investing in a new technology and which patient groups will lose these QALYs, may be a way forward to make opportunity costs more explicit (18). For example, in the case of Orkambi®, a high-priced drug for the treatment of cystic fibrosis, which posed a challenge for public payers, the calculator showed that spending money for this drug results in a net loss of QALYs, displacing healthcare in neurology, ophthalmology, dermatology, and ear diseases. Although resting on efficiency principles, this approach contributes to increasing transparency of equity impacts from reimbursement decisions. Since the Austrian system can be characterized as strongly focusing on need and less on efficiency (7), making equity an important decision criterion, this approach may be of higher value for decision-makers than presenting ICERs. Demonstrating the impact of a new product on the overall system and other patients may help justify restricted access or lower prices to patient advocates, the industry or the media, who often use the unmet needs argument to argue for immediate and unrestricted access and high prices of new products. However, developing an Austrian calculator requires a feasibility study and substantial preparatory work, for example, to generate empirical data to populate the calculator.

### A broader variety of health economic methods is needed to address the scope of technologies

Most country-specific guidelines on economic methods within HTA recommend CUA as the main type of analysis to perform an economic evaluation and specify QALYs as the preferred outcome indicator (19–21). Although the methods defined in the guidelines are primarily intended for evaluating pharmaceuticals (19), they have been applied to all other types of interventions. These include medical devices, diagnostic interventions, or preventive and health-promoting activities. In contrast to deciding on the method based on the nature of an intervention and the research question, we are observing a move toward a “one-size-fits-all approach.”

The AIHTA’s core profile includes evaluating medical devices and public health interventions, many of which fulfill the definition of complex interventions (22). We expect an increase in the number of complex technologies that we will undergo HTA in the future. With the restriction to CUA, we see a risk of producing biased results, which are misleading to decision-makers and may systematically discriminate certain types of interventions or disease groups against others. Some interventions we are dealing with in our work do not primarily aim to improve health-related quality of life (HRQoL) or life expectancy, but have other key intentions. Examples are interventions to prevent the negative consequences of parental mental illness in children or community nursing programs. The primary aim of the former is, for example, to increase children’s knowledge of mental illness, empower them to seek help, improve their social support or self-efficacy, and enhance parenting skills (23). Although some of those outcomes may result in better mental health in the long run, HRQoL, as it is currently assessed (via five dimensions of the EQ-5D), likely does not capture the nature of the core outcomes of those interventions. In community nursing, core outcomes of interest may be social participation, better health literacy or support seeking by carers which are also not represented in standard tools.

Moreover, outcomes in these interventions are often multidimensional, including possible medium-term effects beyond the healthcare sector, such as school attendance and long-term impacts, such as income or educational attainment (24). Furthermore, interventions often aim to improve outcomes in multiple groups, such as children and parents in family focused mental health interventions or patients and carers in community-nursing interventions. Yet, with CUA, outcomes are usually measured only in one group (25;26) and measurement is restricted to a single parameter.

As for benefits, on the cost side, considering intersectoral costs has been emphasized in methodological research (27–29). In practice, many economic evaluations within HTA do not fully cover all relevant intersectoral costs, such as costs for informal care (25;26). More recently, attention has been drawn to environmental costs arising from healthcare interventions (30). Research activities on methods to capture environmental costs are currently taking place (31;32); however, methodological standards on integrating environmental costs into economic evaluations within HTA are currently missing (33). If economic evaluation gains more importance in Austria in the future, we consider it important to fully account for intersectoral costs in economic evaluation in Austrian HTAs and to advocate for considering new developments regarding environmental costs.

As a methodological orientation for Austria, we suggest broader cost-effectiveness frameworks developed in methodological research (17), and guidelines for evaluating complex interventions that intend to overcome some of the current shortcomings (22). Approaches recommended in those documents that we find useful include developing a logic model for economic evaluation outlining the mechanisms by which an intervention is thought to generate outcomes. This provides a rationale for the costs and outcomes to be selected for the economic evaluation. Another recommendation we support when evaluating complex interventions is to broaden the spectrum of evaluation types to cost-consequence analysis as a method that gives room to present more than one outcome and thus more likely captures the full range of costs and consequences from an intervention. Another promising field under development is realist economic evaluation methods. These aim to merge realist evaluation proposed for complex interventions (guided by the questions “what works for whom under which circumstances?”) and economic evaluation methods (34). Regarding outcomes for economic evaluations, we suggest considering alternatives to QALYs based on EQ-5D, such as approaches based on Sen’s capability approach (35;36), which have been validated and applied in research but less in economic evaluations within HTA, and to monitor and possibly adopt more fundamental developments, such as measures capturing well-being (37). Although those approaches introduce more complexity to the evaluation, have their own limitations, and require more effort to develop study designs, they likely better capture the nature of some interventions than CUA. Not least, the limitations of standard approaches and ways to overcome have long been addressed in the methods for economic evaluations in public health (38–40) and need to be given greater consideration in the Austrian context.

### Applying health economic methods requires a reflection on their underlying values

Although economic evaluation may at first sight seem to be a matter of mathematics and technique, the methods are not “value-neutral.” Health economists rarely discuss that the economic evaluation methods they apply and propose for healthcare decision-making are fundamentally rooted in neoclassical economic theory and represent the ethical principle of utilitarianism (21). Presenting methods without underlying assumptions has been termed “collective scientific unconsciousness” or “intellectual bias” (41). The underlying value of utilitarianism is that it defines an action as morally right if it maximizes the aggregated total benefit, that is, the sum of the well-being of all those affected (42). Decision-makers and the society they represent are not automatically aware of these underlying principles, which have substantial implications on how the allocation of resources is prioritized.

Applied in health care, the utilitarian principle of maximizing health or social welfare in economic evaluations may discriminate certain population groups against others as there can be trade-offs between efficiency and equity. On the other hand, the objective of reducing health inequality may clash with the objective of improving total health, for example, when delivering services effectively to socially disadvantaged communities requires additional resources (43). In this case, it could simply be more efficient (and health maximizing) to use those resources to improve the health of less disadvantaged people.

In Austria, it has neither been discussed politically nor ethically, nor have the citizens been asked whether or in which situations maximizing health gains should be a key priority of reimbursement decisions, considering the potential consequences of increasing health inequalities. Furthermore, there is no legal analysis of which methods comply with the Austrian healthcare legislation, how much leeway there is for regulation, and whether methodological details would violate the legal foundations or require law changes. For example, in Germany, the legislation was the main reason why the initial method for economic evaluation (though never routinely applied) markedly differed from other countries. Legislative changes were also the core driver for later revisions. Additionally, a discussion in the German ethics council preceded method decisions (44;45).

Based on these reflections, the development of health economics methods to be applied in HTA in Austria and the generation of methodological guidelines need to go hand in hand with a reflection on societal values. The methods researchers are selecting within an HTA need to follow those values rather than copying methods without considering their underlying moral principles. Such a discussion could also help to increase transparency in the decision-making process and improve accountability for reasonableness more broadly (46). For example, if a key priority in Austria is to reduce health inequalities, distributional cost-effectiveness analysis or extended cost-effectiveness analysis (47) may play an important role in future. On the contrary, when maximizing population health is agreed upon as the top priority, the traditional methods of CUA or CEA may be relevant, taking, however, into account their limitations outlined earlier. Several HTA agencies have invested in eliciting societal preferences to define equity weights, for example, regarding disease severity or rarity. They are applied to adjust cost-effectiveness ratios and thresholds or in other ways in the reimbursement decision processes (48). These can serve as a useful starting point for Austria. Yet, values we as a society want to follow are political, not scientific decisions, requiring a more transparent political and societal discourse about them. Researchers can and should, however, support this decision by collecting robust empirical data on societal values and citizen’s views on criteria that should be applied in reimbursement decisions in health care. Researchers must also inform decision-makers about the values behind the different health economics methods rather than presenting them to them as “neutral” mathematical methods and statistical techniques. According to Lessard (41), awareness of the ethical dimensions of economic evaluation requires a commitment to reflexivity among health economists. This will provide a basis for the acceptance of economic evidence at the policy level in Austria. Only then can lawyers and administrations make sure that the methods to be applied for informing healthcare decisions are in line with legal requirements; and only then can they be specified in a health economic guideline to be followed by researchers or the industry when conducting economic studies.

### Health economics within HTA needs to go beyond microeconomic analysis of interventions

Since the beginning of supporting decisions with HTA, there have been endeavors at national and supranational levels (e.g., EUnetHTA) to improve processes and methods. Despite these efforts, the sustainability of our public healthcare systems is more at risk than ever. It seems HTA is mostly alleviating symptoms caused somewhere else. It does not offer solutions to address the origin of the problems.

One reason for this development is in our view that HTA is primarily reactively responding on the microlevel to the supply of products defined by the industry based on profit potentials. In addition to the sound evaluation of individual products, we, therefore, see a need for Austrian HTA experts to engage at the policy level. For health economics within HTA in Austria, this means contributing to a better understanding of economic policies such as industrial policies, pricing policies, intellectual property rights, and so forth, and to engage in discussions and further develop such policies and laws. In contrast to natural laws, these laws have been formulated by humans. They are, therefore, not set in stone and can be changed.

In addition to informing decision-makers on the economic dimensions of single interventions, we see our responsibility in raising awareness on the dynamics of the pharma and med-tech market and reducing information asymmetry between the industry and public healthcare administrations by disentangling complexity and improving economic literacy. An example is our engagement in the Horizon Europe project “HI-PRIX,” in which we are aiming to enhance the understanding of what drives the costs of developing new pharmaceuticals, what are the public contributions in those processes and how this information may be used for pricing in future (31).

This is to be seen in light of the urgent necessity of new economic thinking. As Mariana Mazzucato, the head of the WHO Council on the Economics of Health for All, states, “States can move away from reactively fixing market failures to proactively and collaboratively shaping markets that prioritize human and planetary health” (49, p. iv). As one of the main fields of action, the WHO Council report suggests re-organizing public financing and governance toward health and health determinants, thereby establishing cross-sectoral collaboration across all ministries and goal-oriented public funding processes. Moreover, the WHO Council proposes a mission-oriented industry strategy, meaning to identify public health needs, support research accordingly, and set conditionalities if public resources contribute to the development of new technologies (e.g., regarding access, prices or property rights) (49). Several initiatives indicate that the move toward those priorities has started, such as the reform of the EU pharmaceutical legislation (50) or a Belgian project that aims to identify and measure unmet health-related needs for a more needs-driven healthcare policy and innovation development (51). Our understanding of health economics within HTA in Austria is to engage in or initiate such projects and invest in efforts to transfer the knowledge gained to decision-makers. In addition, we see it as our responsibility to reflect on what new economic concepts discussed in the context of planetary health at the societal level (49) mean for health economic methods in HTA, which currently reflect the “old” economic paradigm both linguistically and conceptually (e.g., the mindset that a healthy population primarily serves as a productivity factor and human capital for a thriving economy in contrast to emerging concepts that see a need to reorganize the economic system in such a way that it is in service for human and planetary health).

## Concluding remarks

Health economics expertise will be vital in meeting current challenges regarding the sustainability of a public healthcare system in Austria. Although health economic methods in the form of economic evaluations have been an integral and formal part of HTA in many countries, they have played a negligible role in Austria. In this commentary, we argue that health economics evidence needs to be more routinely integrated into health policymaking in Austria. However, we believe that the methodological scope currently observed in HTA internationally needs to be broadened in Austria to better address the nature of interventions and decision-makers’ needs and to avoid misleading results from following a one-size-fits-all approach. We also propose to be more transparent on the values underlying the methods and to reflect their application in the context of Austrian law and societal values. Finally, we argue that the current focus on microeconomic methods within HTA needs to be embedded within the broader macrolevel of a health and economic system. We suggest considering several alternative methods, encouraging out-of-the-box thinking, and experimenting with new approaches. The recent developments for evaluating drugs in the hospital setting will be an opportunity to discuss the relevance of economic evidence. They may be a chance to implement some of the approaches suggested for Austria and to improve the understanding of the benefits and limitations of different methods among decision-makers. They may also demonstrate the need for a method guideline and research activities that are needed in parallel to support the guideline. Therefore, with this article, we hope to stimulate discussion among Austrian decision-makers, HTA units and methods experts from the academic field. However, while some of our topics are unique for Austria, many challenges we address equally apply to other healthcare systems. Our thoughts may then be relevant not just for Austria but for further developing health economics within HTA internationally. To use new methods routinely within HTA will require substantial methodological research and collection of empirical data both in Austria and elsewhere. Health economists within HTA and in academia need to collaborate to engage in this endeavor successfully.
